# Permeability Prediction in Deep Coal Seam: A Case Study on the No. 3 Coal Seam of the Southern Qinshui Basin in China

**DOI:** 10.1155/2013/161457

**Published:** 2013-12-12

**Authors:** Pinkun Guo, Yuanping Cheng

**Affiliations:** ^1^School of Safety Engineering, China University of Mining & Technology, Xuzhou 221116, China; ^2^National Engineering Research Center for Coal & Gas Control, China University of Mining & Technology, Xuzhou 221116, China

## Abstract

The coal permeability is an important parameter in mine methane control and coal bed methane (CBM) exploitation, which determines the practicability of methane extraction. Permeability prediction in deep coal seam plays a significant role in evaluating the practicability of CBM exploitation. The coal permeability depends on the coal fractures controlled by strata stress, gas pressure, and strata temperature which change with depth. The effect of the strata stress, gas pressure, and strata temperature on the coal (the coal matrix and fracture) under triaxial stress and strain conditions was studied. Then we got the change of coal porosity with strata stress, gas pressure, and strata temperature and established a coal permeability model under tri-axial stress and strain conditions. The permeability of the No. 3 coal seam of the Southern Qinshui Basin in China was predicted, which is consistent with that tested in the field. The effect of the sorption swelling on porosity (permeability) firstly increases rapidly and then slowly with the increase of depth. However, the effect of thermal expansion and effective stress compression on porosity (permeability) increases linearly with the increase of depth. The most effective way to improve the permeability in exploiting CBM or extracting methane is to reduce the effective stress.

## 1. Introduction

Coal bed methane (CBM) is a natural product in the coalification process [1, 2]. CBM is a serious threat to safety in underground coal mining and can cause disasters, such as coal and gas outbursts and gas explosions [1, 3]. However, CBM is also an unconventional natural gas resource that has been exploited worldwide in countries such as USA, Australia, and China [4, 5].

Coal permeability is an important parameter in mine methane control and CBM exploitation because it determines the practicability of methane extraction. The permeability determined in the laboratory is not the real value of coal seam tested in the field. The coal seam permeability is completed based on the well logs in the CBM exploitation and is calculated based on the gas flow theory of the hole in or through the coal seam during coal mining [2, 6]. In the region where the exploration or mining does not conduct, especially in deep coal seam, it requires a large number of engineering works to test the permeability, which is very expensive. The permeability of deep coal seam predicted by a permeability model is fast, reliable, and economical for preassessment of CBM exploitation and methane extraction.

The permeability of coal depends on the fracture characteristics, including the size, spacing, connectivity, width, mineral fill, and distribution [7], which are affected by the strata stress, gas pressure, and temperature of the coal seam.

Several models have been proposed to explain the variability of coal permeability [8–19]. The coal permeability models can be divided into two important classes [4]: those under uniaxial strain conditions [8–13] and those under triaxial stress conditions [14–19].

However, uniaxial strain conditions are a simplified homogenisation of the stress-strain states of coal during mining and exploitation and may be valid at the scale of a relatively large basin; the mechanical conditions at the local scale are expected to be much more complex in coal seams [16]. And the tri-axial strata stress conditions change with depth. Therefore, it is appropriate for predicting the permeability of deep coal seam using a permeability model under conditions of tri-axial stress-strain.

The effects of effective stress and matrix sorption deformation are considered by most coal permeability models [8, 9, 11–19] except for Seide and Huitt's model who assumed that cleat deformation was caused entirely by desorption shrinkage [10]. The coal matrix is assumed to be incompressible by assuming that the bulk modulus of the coal matrix is much larger than the coal bulk modulus and then Biot's coefficient *α* is assumed to be 1 [8–13, 16, 17]. However, the compression of the coal matrix by the pore pressure could not be ignored [20, 21]. Therefore, the Biot's coefficient for coal is less than 1 [17, 22, 23].

Most models consider the matrix deformation to be equal to the fracture deformation. However, only part of the matrix deformation contributes to the fracture deformation [24]. Liu and Rutqvist [16] and Connell et al. [17] established permeability models in which the sorption deformation applied to the fracture.

The strata stress, gas pressure, and temperature of the coal seam change with the depth [25–29]. As a result, the permeability of the coal seam changes with depth. In this paper, we discussed the effect of stress, gas pressure, and temperature on the porosity of the coal (the coal matrix and the fracture) under tri-axial stress-strain conditions. Then we established a permeability model for deep coal seam. At last, we predicted the permeability of the No. 3 coal seam of South Qinshui Basin (SQB) in China.

## 2. Establishment of the Permeability Model

**Figure 1:**
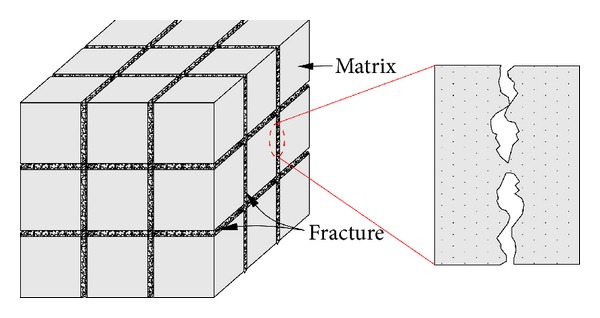
Schematic of coal structure.

The coal has a natural dual porosity structure that consists of the coal matrix and the fracture in which there are numerous inorganic minerals, mainly kaolinite, pyrite, and illite, as shown in Figure 1. More than 95% of the gas occurs as adsorbed gas in the sorption space of the abundant micropores [8]. The gas migrates by diffusion in the micropore system and follows Fick's Law. The closely spaced natural fractures surrounding the coal matrix, which form the cleat system, determine the mechanical properties of the coal and the flow paths for the methane; this flow follows Darcy's Law. Therefore, the coal fracture permeability is closely related to the characteristics of the fractures, which are controlled by the coal rank, geologic structure, mining, strata stress, gas pressure, formation temperature, and other factors. The coal fracture porosity is controlled by the strata stress, gas pressure, and temperature of the coal seam which change with the depth. Therefore, we analyzed the effect of the strata stress, gas pressure, and temperature of the coal seam on the fracture in the later section.

Before analysis, we make the following assumptions.

1. Coal is considered to be a dual continuous isotropic elastic medium even though the coal consists of coal matrix and fracture.
2. The strain is elastic and infinitesimal, so the second and higher order terms can be ignored. Therefore, the strains induced by different factors can be added.
3. The effect of methane on the coal is divided into normal effective stress effect of the gas like nonadsorptive gas and the effect of sorption deformation, which are added directly.

### 2.1. Effective Stress

According to the effective stress principle [30], the bulk volumetric strain increment and the pore volume strain increment can be expressed as [19, 31]

(1) $$\begin{array}{c} d\varepsilon_{e}=\frac{dV_{e}}{V}=-\frac{1}{K}\left(d\overset{-}{\sigma }-\alpha dp\right), \end{array}$$

(2) $$\begin{array}{c} d\varepsilon_{ep}=\frac{dV_{ep}}{V_{p}}=-\frac{1}{K_{p}}\left(d\overset{-}{\sigma }-\beta dp\right), \end{array}$$

where *ε*_*e*_ and *V*_*e*_ are the coal bulk strain and volume caused by the effective stress, respectively; *ε*_*ep*_ and *V*_*ep*_ are the pore strain and volume caused by the effective stress, respectively; $\overset{-}{\sigma }=(1/3)(\sigma_{11}+\sigma_{22}+\sigma_{33})$ is the mean stress, MPa; *K* = *E*/3(1 − 2*v*) is the coal bulk modulus, MPa; *K*_*p*_ is the coal pore modulus, MPa; *α* = 1 − *K*/*K*_*m*_ is Biot's coefficient; *β* = 1 − *K*_*p*_/*K*_*m*_ is the effective coefficient for the pore system; *K*_*m*_ is the coal matrix modulus, MPa; *E* is the elastic modulus of the coal, MPa; and *v* is Poisson's ratio.

Without the gas sorption effect, the volumetric change of the porous medium satisfies the Betti-Maxwell reciprocal theorem ${\left(\partial V/\partial p\right)}_{\overset{-}{\sigma }}={\left(\partial V_{p}/\partial \overset{-}{\sigma }\right)}_{p}$, and we obtain

(3) $$\begin{array}{c} K_{p}=\frac{\phi }{\alpha }K, \end{array}$$

where *ϕ* is the porosity.

### 2.2. The Coal Matrix Deformation

The coal matrix swells due to adsorb gas and temperature increase underground.

*(1) Thermal Deformation*. The deformation of the coal matrix due to temperature change is expressed as

(4) $$\begin{array}{c} d\varepsilon_{mt}=\eta dT, \end{array}$$

where *ε*_*mt*_ is the thermal deformation of the coal matrix; *η* is the coefficient of the thermal deformation, K^−1^; *T* is the temperature of the strata, K.

*(2) Sorption Deformation*. The gas sorption capacity of coal increases with pressure and closely follows Langmuir type isotherm. Langmuir isotherm describes the amount of adsorbed gas on the coal as a function of pressure and has the following form [32]:

(5) $$\begin{array}{c} V_{ad}=\frac{abp}{1+bp}, \end{array}$$

where *V*_*ad*_ is the adsorbed gas volume, m^3^/t; *a* is the Langmuir volume, m^3^/t; *b* is the adsorption coefficient, MPa^−1^.

Adsorption coefficient indicates coal's ability to adsorb gases and is exponentially increased with the energy of adsorption and decreased with the temperature of adsorption. Adsorption coefficient in a Langmuir-type isotherm can be estimated using the Arrhenius rate equation at equilibrium condition, in which affinity is a function of temperature and is given by the following equation [33]:

(6) $$\begin{array}{c} b=\frac{b_{0}}{\sqrt{T}}\exp\left(\frac{\Delta H}{RT}\right),\\ b_{0}=\frac{s_{\alpha }}{k_{d\infty }\sqrt{2\pi MR}}, \end{array}$$

where Δ*H* is the heat of sorption, J/mol; *R* is the gas universal constant, J/mol·K.

This is to say that the adsorption capacity is independent of temperature, and as a result the heat of adsorption is a constant, independent of loading. Increase in the temperature will decrease the adsorbed amount at a given pressure. This is due to the greater energy acquired by the adsorbed molecule to evaporate.

It has been tested and verified that the coal swells it when adsorbing gas and the adsorption deformation is proportional to the adsorbed gas volume [22, 24, 34]:

(7) $$\begin{array}{c} \varepsilon_{s}=\chi V_{ad}=\chi \frac{abp}{1+bp}, \end{array}$$

where *ε*_*s*_ is the sorption deformation of the coal matrix; *χ* is the coefficient of sorption deformation, t/m^3^.

However, the coal matrix is also compressed by the sorptive gas in addition to swelling due to adsorption [20, 21, 35]. When testing the sorption deformation, we need the following equation to calibrate the experimental data:

(8) $$\begin{array}{c} \varepsilon_{s}=\varepsilon_{\exp}+\frac{p}{K_{s}}, \end{array}$$

where *ε*_exp⁡_ is the deformation measured in the experiment.

Therefore, the coal matrix deformation is the sum of adsorption deformation and thermal deformation:

(9) $$\begin{array}{c} \varepsilon_{m}=\varepsilon_{s}+\varepsilon_{T}. \end{array}$$

*(3) Fracture Deformation Caused by the Coal Matrix*. Deformation of the coal matrix can affect the deformation of both the bulk coal and the fractures in the coal [10, 11, 14, 36]. The coal matrix deformation is assumed to contribute entirely to the fracture deformation [10, 11, 15]. However, the contribution of coal matrix deformation to the fracture has been significantly overestimated [16, 17, 24]. For example, Roberston and Christiansen [24] demonstrated that the most commonly used models [11, 12] significantly overestimate the effects of matrix swelling on the permeability changes which was observed in laboratory experiments.

The coal is divided into the matrixes by the fracture, but there are bridges between the matrixes, as shown in Figure 1. When the coal matrix swells, the bridge limits the coal matrix deformation to the fracture [16]. Karacan [37] and Dawson et al. [38] found that numerous inorganic minerals, mainly kaolinite, pyrite, and illite, are present in coal fractures. These minerals prevent the coal matrix from completely closing the fracture. Therefore, only part of the matrix deformation contributes to the fracture deformation.

The effective coal matrix deformation factor, *f*_*m*_, is introduced to measure the degree of influence of the coal matrix deformation on fracture deformation. The factor *f*_*m*_ is a parameter of the coal structure and depends on the distribution of fractures, the filling characteristics of the fracture, and other factors. For a particular coal, *f*_*m*_ is a constant between 0 and 1. If there is no fracture in the coal, the parameter *f*_*m*_ is equal to 0. The parameter *f*_*m*_ would be equal to 1 when two surfaces of the fracture are smooth and parallel.

Thus, the fracture deformation due to the coal matrix deformation is expressed as

(10) $$\begin{array}{c} dV_{mf}=f_{m}dV_{m}=f_{m}V_{m}\left(d\varepsilon_{s}+d\varepsilon_{T}\right), \end{array}$$

where *V*_*mf*_ is the fracture volume deformation due to deformation of coal matrix; *V*_*m*_ is the volume of coal matrix.

### 2.3. The Permeability Model under Triaxial Stress Conditions

As a porous medium, the coal bulk volume *V* is composed of the matrix volume *V*_*m*_ and the pore volume *V*_*p*_:

(11) $$\begin{array}{c} V=V_{p}+V_{m}. \end{array}$$

Based on the definition of porosity, *ϕ* = *V*_*p*_/*V*, we obtain

(12) $$\begin{array}{c} d\phi =d\left(\frac{V_{p}}{V}\right)=\phi \left(\frac{dV_{p}}{V_{p}}-\frac{dV}{V}\right). \end{array}$$

The bulk volume deformation of coal is equal to the sum of the deformation due to the effective stress and the coal matrix deformation due to adsorption and temperature change:

(13) $$\begin{array}{c} dV=dV_{e}+dV_{mv}=-\frac{V}{K}\left(d\overset{-}{\sigma }-\alpha dp\right)+\left(1-f_{m}\right)V_{m}d\varepsilon_{m}, \end{array}$$

where *V*_*mv*_ is the bulk volume deformation due to the matrix deformation.

Dividing both sides of (13) by the coal bulk volume, we obtain

(14) $$\begin{array}{c} \frac{dV}{V}=-\frac{1}{K}\left(d\overset{-}{\sigma }-\alpha dp\right)+\left(1-f_{m}\right)\left(1-\phi \right)d\varepsilon_{m}. \end{array}$$

Similarly, from (2) and (10), we obtain

(15) $$\begin{array}{c} \frac{dV_{p}}{V_{p}}=-\frac{1}{K_{p}}\left(d\overset{-}{\sigma }-\beta dp\right)-\frac{1-\phi }{\phi }f_{m}d\varepsilon_{m}. \end{array}$$

By substituting (14) and (15) into (12), we obtain

(16) dϕϕ=−1Kp(σ−−βp)+1K(σ−−αp)    −(1−ϕϕfm+(1−fm)(1−ϕ))dεm.

Then, substituting *K*_*p*_ = (*ϕ*/*α*)*K* and *β* = 1 − *K*_*p*_/*K*_*m*_ into (16) and considering that *ϕ* ≪ 1 (*ϕ* < 10%), we can rearrange and simplify the equation to obtain

(17) $$\begin{array}{c} d\phi =-\frac{\alpha }{K}\left(d\overset{-}{\sigma }-dp\right)-f_{m}d\varepsilon_{m}. \end{array}$$

Integrating (17) gives

(18) ϕ=ϕ0−αK[(σ−−σ−0)−(p−p0)]  −fm[χ(abp1+bp−ab0p01+b0p0)+η(T−T0)].

The widely used cubic relationship between permeability and porosity [8–17] is given

(19) $$\begin{array}{c} \frac{k}{k_{0}}={\left(\frac{\phi }{\phi_{0}}\right)}^{3}, \end{array}$$

where *k* is the coal permeability.

Substituting (18) into (19), the coal permeability model that considers the effect of the effective stress and coal matrix deformation (ESMD model) is given

(20) kk0={1−αϕ0K[(σ−−σ−0)−(p−p0)]  −fmϕ0[χ(abp1+bp−ab0p01+b0p0)+η(T−T0)]}3.

It is clear that the model contains an effective stress term and a coal matrix deformation term. The factor *f*_*m*_ measures the degree of influence of the coal matrix deformation on the fracture deformation.

## 3. Model Validation and Evaluation

### 3.1. Experimental Data

Numerous laboratory experiments have been conducted on coal permeability [24, 39, 40]. Pini et al. [40] conducted experiments that tested the mechanical parameters, porosity, adsorption swelling parameters, and coal permeability of a coal sample (Sulcis coal sample) from the Monte Sinni coal mine in the Sulcis Coal Province (Sardinia, Italy). We use the experimental data to validate and evaluate the ESMD model because of the comprehensive set of parameters available for the coal sample and the detailed experimental data. The coal permeability experiments were conducted under hydrostatic conditions at a constant confining pressure (10 MPa) and various gas pressures between 0 MPa and 8 MPa at 45°C using N_2_ and CO_2_. The adsorption swelling parameters of the Sulcis coal sample for N_2_ and CO_2_ were corrected using (8). The parameters of the Sulcis coal sample are shown in Table 1.

### 3.2. Validation

The experimental data were matched by the ESMD model, the Palmer-Mansoori (P-M) model [11], the Shi-Durucan (S-D) model [12], and the Robertson-Christiansen (R-C) model [14] using the parameters in Table 1. The results are shown in Figure 3. The ESMD model can predict the experimental data well.

Only part of the matrix deformation contributes to the fracture deformation. The factor *f*_*m*_, which ranges from 0 to 1, is introduced to measure the degree of influence of the coal matrix deformation on the fracture deformation in the ESMD model. The factor *f*_*m*_ is a parameter of the coal structure and does not vary with the type of gas. The factor *f*_*m*_ of the Sulcis coal sample is 0.1723 for both N_2_ and CO_2_. Biot's coefficient *α* of coal is less than 1, and *α* = 0.925 for the Sulcis coal sample, which has a bulk modulus of 778 MPa and a matrix modulus of 10,340 MPa.

**Figure 2:**
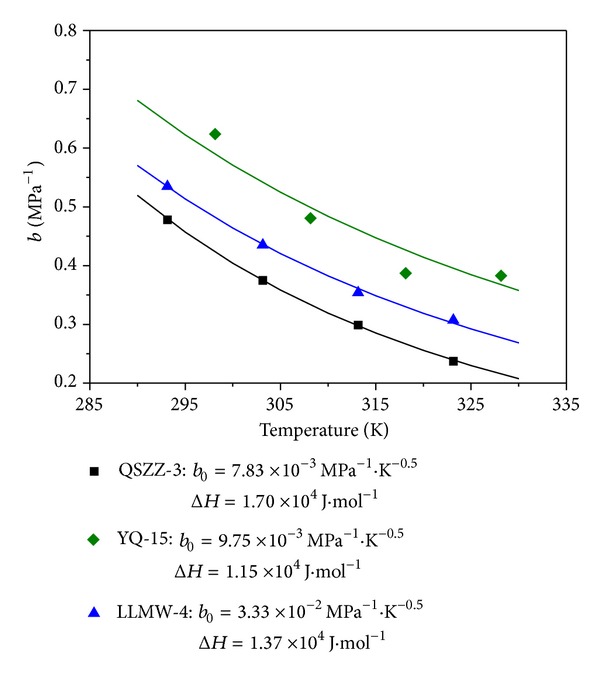
Relationship between the adsorption parameter and temperature.

**Figure 3:**
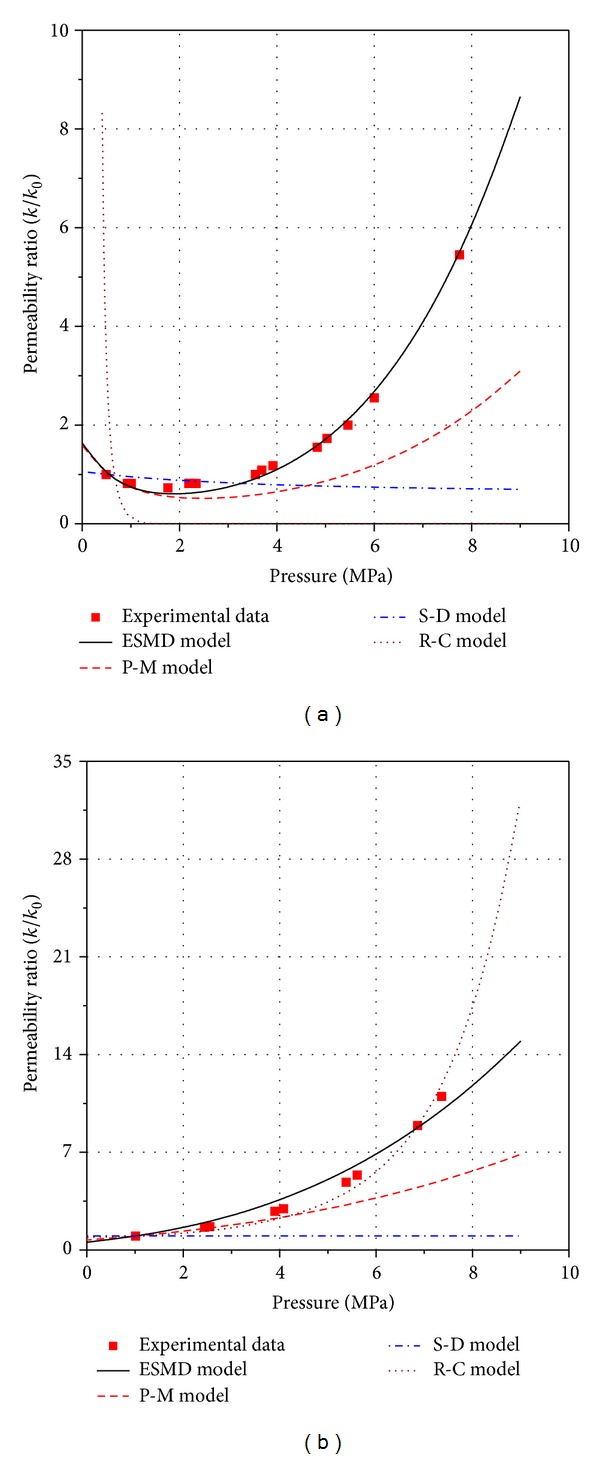
The comparison between permeability models and the experimental data.

The three models poorly match the experimental data for two reasons: in all three models, Biot's coefficient is assumed to be 1 in the P-M model and the S-D model by assuming that the coal matrix is incompressible. Deformation of the coal matrix contributes to the fracture deformation entirely in the three models, which is an overestimation. As shown in Figure 2, the R-C model matches the experimental data well for N_2_ but poorly for CO_2_. The decline rate of fracture compressibility with increasing effective stress *θ* for the R-C model is 2.65 × 10^−14^ MPa^−1^, which implies that the fracture compressibility does not vary with the effective stress. However, the decline rate *θ* varies between 2.45 × 10^−2^ MPa^−1^ and 2.61 × 10^−1^ MPa^−1^ [14, 24, 41].

## 4. The Parameters of the No. 3 Coal Seam in the SQB

The SQB has become the China's first commercial CBM reservoir. CBM reservoir is an unconventional gas reservoir and located at shallow depths, compared to the conventional gas reservoir. The SQB refers to a region, including Changzhi, Gaoping, Jincheng, Yangcheng, Qinshui, and Anze in the southeast of Shanxi Province. It is the most important production base for high quality anthracite in China. Coal seams, generated in Carboniferous and Permian periods, contain abundant methane. Permeability in the coalbed reservoir is relatively high compared to other CBM reservoirs in China. The exploration and production tests in this field have been conducted since 1990s. The results show that the Qinshui Basin is a very promising coalbed methane reservoir with the most exploration wells, the best development prospect, and a higher commercialized production in China's CBM reservoirs. The No. 3 coal seam is the major coal seam mined and CBM reservoir in the SQB. The thickness of No. 3 coal seam in the SQB changes from 0.5 m to 7.0 m with an average value of 5.5 m. The maximum depth is about 2000 m. The coal is anthracite mainly. In this paper, we predicted the permeability of the No. 3 coal seam.

### 4.1. Gas Pressure

**Figure 4:**
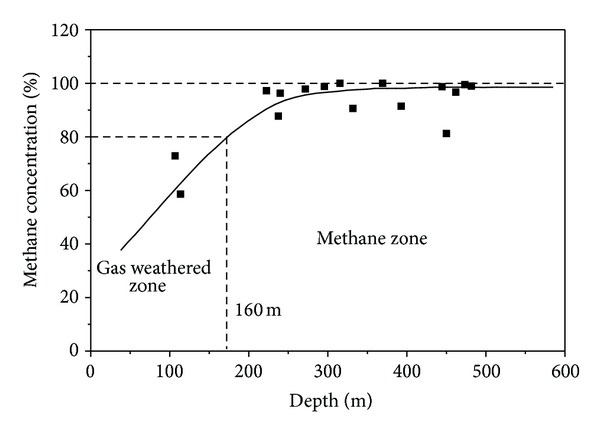
The methane concentration change of the No. 3 coal seam in the SQB with the depth.

There are two different directions of gas migration in the coal seam with an outcrop. For example, the generated methane migrates upward and the surface air migrates downward in the coal seam. Thus, there are two vertical zones of the CBM occurrence in the coal seam, which are the gas weathered zone and the methane zone [27]. Generally, they are zoned by the methane concentration of the coal seam with the value of 80%. The methane concentration change of the No. 3 coal seam in the SQB with the depth is present in Figure 4 [42]. The depth of the gas weathered zone of the No. 3 coal seam is about 160 m.

**Figure 5:**
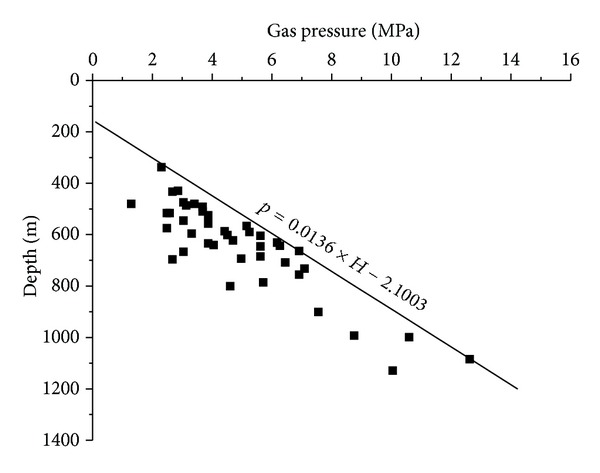
The relationship between gas pressure and depth of No. 3 coal seam in the SQB.

The gas pressure of the No. 3 coal seam in the SQB is present in Figure 5. It has been found that the relationship between gas pressure and depth is linear by analyzing the numerous measured gas pressure values [1, 2, 26–28]. There are many factors that influence the gas pressure which could lead to a deviation from actual values, and the measured data do not possess basic conditions for regression methods [27, 28]. A pressure prediction method, the safety line method, was used to analyse the variation of the gas pressure with depth [28]. Two true symbol points were selected to make the safety line. All the other points except the abnormal points are below the line, as shown in Figure 5. The gas pressure gradient is 0.0136 MPa/m. The relationship between the gas pressure and the depth is expressed as

(21) $$\begin{array}{c} p=0.0136\times H-2.1003, \end{array}$$

where *H* is the depth of coal seam, *m*.

The gas pressure calculated by (21) at the depth of 161 m is 0.1 MPa. The result is consistent with the depth of the gas weathered zone because the gas pressure at the depth of the gas weathered zone is from 0.1 to 0.15 MPa statistically in China [1, 27].

### 4.2. The Strata Stress

It has been practically verified that the strata stress increases linearly with depth [25, 43]. Meng et al. [26] investigated the stratum stresses in the SQB and they are expressed as

(22) $$\begin{array}{c} \sigma_{V}=0.027\times H,\\ \sigma_{H}=0.0304\times H-3.1975,\\ \sigma_{h}=0.0235\times H-3.5127, \end{array}$$

where *σ*_*V*_ is the vertical stress, MPa; *σ*_*H*_ is the maximum horizontal stress, MPa; *σ*_*h*_ is the minimum horizontal stress, MPa.

### 4.3. The Strata Temperature

The temperature of the strata also increases linearly with the depth. Sun et al. [29] investigated the strata temperature in SQB and found that the relationship between the strata temperature and the depth is

(23) $$\begin{array}{c} T=G\times \left(H-20\right)+282.15. \end{array}$$

### 4.4. The Parameters of No. 3 Coal Seam

The parameters of the No. 3 coal seam in the SQB have been measured by many researchers [26, 42, 44–47]. The parameters on average are presented in Table 2. Bangham and Franklin [48] and Kelemen and Kwiatek [49] measured the thermal deformation coefficient of different coal samples at the temperature less than 100°C. The thermal deformation coefficient was 38.13 × 10^−6^ K^−1^, which was used in the prediction.

**Figure 6:**
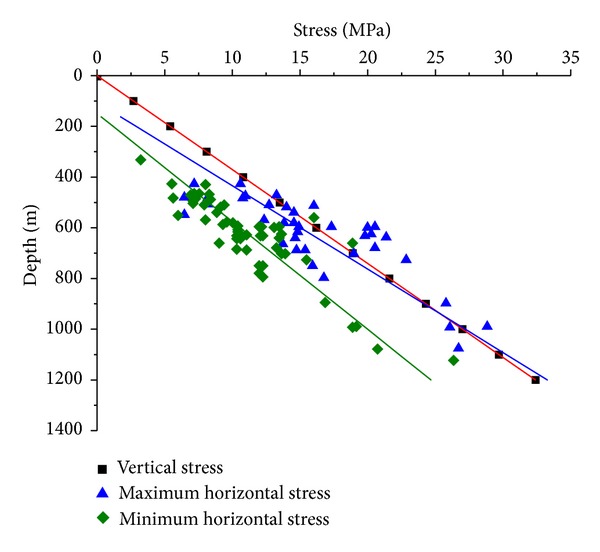
The strata stress in the SQB.

The prediction of the permeability of the No. 3 coal seam in the SQB was conducted by using (6) and (20) to (23) based on the parameters in Table 2. The results were shown in Figure 6.

## 5. Discussions

**Figure 7:**
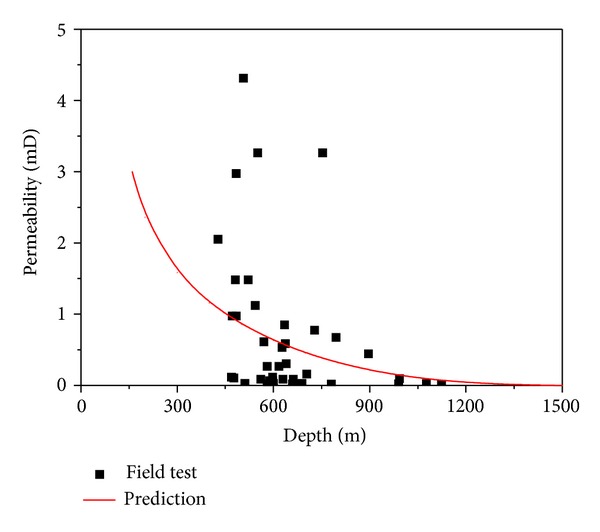
The permeability of No. 3 coal seam in the SQB predicted and tested in field.

**Figure 8:**
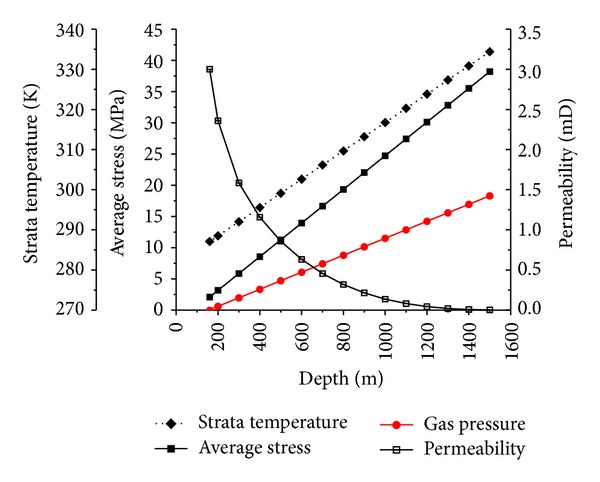
Relationships among strata temperature, average stress, gas pressure, permeability, and the depth of No. 3 coal seam in the SQB.

**Figure 9:**
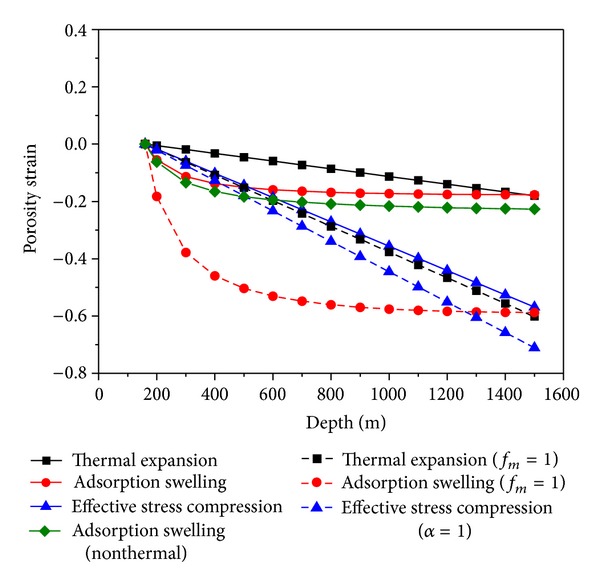
Contributions to the porosity deformation.

(1) The statistics of the permeability of the No. 3 coal seam tested in the field was investigated by Meng et al. [26] and was presented in Figure 7. The permeability model could predict the deep coal permeability of the No.3 coal seam in the SQB efficiently.

(2) The average strata stress, the gas pressure, and the strata temperature of the No. 3 coal seam in the SQB increase linearly with the depth. But the gradient of the average strata stress is larger than that of the gas pressure (Figure 8). So the effective stress also increases with the depth, which compresses and closes the fracture narrower. The fracture is also closed more narrowly by the adsorption swelling and thermal expansion increasing with the depth increase. As a result, the permeability of the No. 3 coal seam in the SQB decreases with the increase of the depth.

(3) There is a cubic relationship between the coal permeability and the coal porosity. The different effects of the effective stress, the adsorption deformation, and the thermal deformation of the coal seam on the permeability could be represented by different effects of them on the porosity which are calculated easily and simply. The different effects of the effective stress, the adsorption deformation, and the thermal deformation were calculated using (18), shown in Figure 9. The negative means that the effect of the facts closes the fracture.

The effect of the adsorption swelling on the porosity increases rapidly at first slowly with the increase of depth. The effect of the thermal expansion and the effective stress compression increases linearly with the depth increase. The effect of the effective stress compression is larger than the others.

As a result, the most effective way to improve the permeability in deep and low permeability coal bed for exploiting CBM or extracting methane is to reduce the effective stress. For example, the method of protective layer mining, which is widely used in China, is very effective and it reduces the stress of the overlying and underlying strata [27].

**Figure 10:**
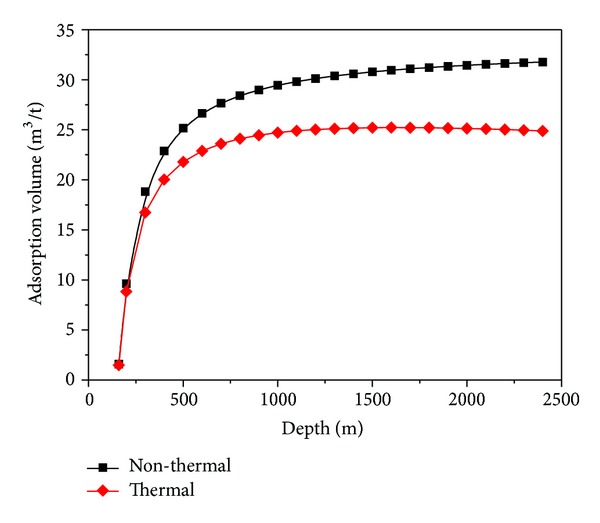
Changes of adsorption volume of the No. 3 coal seam with the depth in the QSB.

(4) The temperature affects the sorption capacity of the coal. The sorption capacity decreases with the increase of the temperature. The adsorption volume increases with the increase of the depth to a maximum value at the depth of 1600 m, and then it decreases. However, if the effect of the temperature on the sorption capacity is neglected, the adsorption volume increases always with the increase of the depth. And the adsorption volume is overestimated (Figure 10). As a result, the effect of the adsorption swelling is also overestimated (Figure 9).

In the ESMD model proposed in this paper, only part of the matrix deformation contributes to the fracture deformation. If the matrix deformation contributes to the fracture deformation, the effect of the matrix deformation due to adsorption swelling and thermal expansion is overestimated significantly (Figure 9).

The Biot's coefficient is less than 1 in the ESMD model, which is true for the coal. But in mangy coal permeability models, it is equal to 1 based on the assumption that the bulk modulus of the coal matrix is much larger than that of the coal, which overestimates the effect of the effective stress (Figure 9).

## 6. Conclusions

The coal permeability is an important parameter in mine methane control and CBM exploitation, which determines the practicability of methane extraction. Permeability prediction in deep coal seam plays a significant role in evaluating the practicability of CBM exploitation.

The coal permeability depends on the coal fractures controlled by strata stress, gas pressure, and strata temperature which change with depth. The effect of the strata stress, gas pressure, and strata temperature on the coal (the coal matrix and fracture) under tri-axial stress and strain conditions was studied. Then we got the change of coal porosity with strata stress, gas pressure, and strata temperature and established a coal permeability model (ESMD model) under tri-axial stress and strain conditions. The coal permeability is controlled by the effective stress, sorption deformation, and thermal expansion. The sorption capacity decreases with the increase of the temperature and thus the sorption deformation is affected by temperature. And only part of the matrix deformation contributes to the fracture deformation. The Biot's coefficient is less than 1 in the ESMD model, which is true for the coal.

The permeability of the No. 3 coal seam of the SQB in China was predicted, which is consistent with that tested in the field.

The effect of the sorption deformation on porosity (permeability) firstly increases rapidly and then slowly with the increase of depth. However, the effect of thermal expansion and effective stress compression on porosity (permeability) increases linearly with the increase of depth. When exploiting CBM or extracting methane, the most effective way to improve the permeability in CBM exploitation or mine methane control is to reduce the effective stress. For example, the method of protective layer mining, which is widely used in China, is very effective, which reduces the stress of the overlying and underlying strata.

## Figures and Tables

**Table 1:** Parameter magnitudes for matching the experimental data.

Parameter	Value
Elastic modulus, *E* (GPa)	1.12
Poisson's ratio, *ν*	0.26
Bulk modulus, *K* (MPa)	778
Matrix modulus, *K*_*m*_ (MPa)	10340
Constrained axial modulus, *M* (MPa)	1369
Biot's coefficient, *α*	0.925
Initial porosity (for N_2_), *ϕ*_0_ (%)	0.585
Initial porosity (for CO_2_), *ϕ*_0_ (%)	0.42
Maximum sorption volume (for N_2_), *a* (m^3^/t)	30.05
Langmuir parameter (for N_2_), *b* (MPa^−1^)	0.07
Sorption swelling coefficient (for N_2_), *χ* (t/m^3^)	4.99 × 10^−4^
Langmuir volume (for CO_2_), *a* (m^3^/t)	55.78
Sorption parameter (for CO_2_), *b* (MPa^−1^)	0.48
Sorption swelling coefficient (for CO_2_), *χ* (t/m^3^)	9.30 × 10^−4^
Effective coal matrix deformation factor, *f*_*m*_	0.1723
Empirical parameter for P-M model, *f*	0.1
Matrix compressibility, *γ* (MPa^−1^)	9.67 × 10^−5^
Fracture compressibility, *C*_*f*_ (MPa^−1^)	0.013
Initial fracture compressibility, *C*_0_ (MPa^−1^)	0.3422
Decline rate of fracture compressibility with increasing effective stress, *θ* (MPa^−1^)	2.65 × 10^−14^

**Table 2:** The parameters for predicting permeability in the SQB.

Parameters	Values
Elastic modulus, *E* (GPa)	3.36
Poisson's ratio, *ν*	0.30
Biot's coefficient, *α*	0.80
Coefficient of the thermal deformation, *η* (K^−1^)	38.13 × 10^−6^
Langmuir volume, *a* (m^3^/t)	31.43
Adsorption heat, Δ*H* (J)	1.70 × 10^4^
Adsorption parameter coefficient, *b*_0_ (MPa^−1^·K^−0.5^)	8.95 × 10^−3^
Sorption swelling coefficient, *χ* (t/m^3^)	2.23 × 10^−4^
Initial permeability, k_0_ (mD)	3.00
Initial porosity, *ϕ*_0_ (%)	0.90
Effective coal matrix deformation factor, *f*_*m*_	0.25

## References

[1] Yu QX (1992). *Mine Gas Prevention and Control*.

[2] Zhou SN, Lin BQ (1997). *The Theory of Gas Flow and Storage in Coal Seams*.

[3] Karacan CÖ, Ruiz FA, Cotè M, Phipps S (2011). Coal mine methane: a review of capture and utilization practices with benefits to mining safety and to greenhouse gas reduction. *International Journal of Coal Geology*.

[4] Liu J, Chen Z, Elsworth D, Qu H, Chen D (2011). Interactions of multiple processes during CBM extraction: a critical review. *International Journal of Coal Geology*.

[5] Moore TA (2012). Coalbed methane: a review. *International Journal of Coal Geology*.

[6] Liu M-J, He X-Q (2004). Optimistic calculation method of gas seepage coefficients. *Journal of the China Coal Society*.

[7] Laubach SE, Marrett RA, Olson IE, Scott AR (1998). Characteristics and origins of coal cleat: a review. *International Journal of Coal Geology*.

[8] Gray I (1987). Reservoir engineering in coal seams. Part 1: the physical process of gas storage and movement in coal seams. *SPE Reservoir Engineering*.

[9] Sawyer WK, Paul GW, Schraufnagel RA In development and application of A 3-D coalbed simulator.

[10] Seidle JP, Huitt LG Experimental measurement of coal matrix shrinkage due to gas desorption and implications for cleat permeability increases.

[11] Palmer I, Mansoori J (1998). How permeability depends on stress and pore pressure in coalbeds: a new model. *SPE Reservoir Engineering*.

[12] Shi JQ, Durucan S (2004). Drawdown induced changes in permeability of coalbeds: a new interpretation of the reservoir response to primary recovery. *Transport in Porous Media*.

[13] Cui X, Bustin RM (2005). Volumetric strain associated with methane desorption and its impact on coalbed gas production from deep coal seams. *AAPG Bulletin*.

[14] Robertson EP, Christiansen RL A permeability model for coal and other fractured, sorptive-elastic media.

[15] Zhang H, Liu J, Elsworth D (2008). How sorption-induced matrix deformation affects gas flow in coal seams: a new FE model. *International Journal of Rock Mechanics and Mining Sciences*.

[16] Liu H-H, Rutqvist J (2010). A new coal-permeability model: internal swelling stress and fracture-matrix interaction. *Transport in Porous Media*.

[17] Connell LD, Lu M, Pan Z (2010). An analytical coal permeability model for tri-axial strain and stress conditions. *International Journal of Coal Geology*.

[18] Liu J, Chen Z, Elsworth D, Miao X, Mao X (2010). Linking gas-sorption induced changes in coal permeability to directional strains through a modulus reduction ratio. *International Journal of Coal Geology*.

[19] Liu J, Chen Z, Elsworth D, Miao X, Mao X (2011). Evolution of coal permeability from stress-controlled to displacement-controlled swelling conditions. *Fuel*.

[20] Pan Z, Connell LD (2007). A theoretical model for gas adsorption-induced coal swelling. *International Journal of Coal Geology*.

[21] Hol S, Spiers CJ (2012). Competition between adsorption-induced swelling and elastic compression of coal at CO_2_ pressures up to 100 MPa. *Journal of the Mechanics and Physics of Solids*.

[22] Durucan S, Ahsanb M, Shia JQ (2009). Matrix shrinkage and swelling characteristics of European coals. *Energy Procedia*.

[23] George J.D. S, Barakat MA (2001). The change in effective stress associated with shrinkage from gas deportation in coal. *International Journal of Coal Geology*.

[24] Robertson EP, Christiansen RL Modeling permeability in coal using sorption-induced strain data.

[25] Kang H, Zhang X, Si L, Wu Y, Gao F (2010). In-situ stress measurements and stress distribution characteristics in underground coal mines in China. *Engineering Geology*.

[26] Meng Z, Zhang J, Wang R (2011). In-situ stress, pore pressure and stress-dependent permeability in the Southern Qinshui Basin. *International Journal of Rock Mechanics and Mining Sciences*.

[27] Cheng YP, Wang HF, Wang L, Zhou HX (2010). *Theories and Engineering Applications on Coal Mine Gas Control*.

[28] Wang L, Cheng Y-P, Wang L, Guo P-K, Li W (2012). Safety line method for the prediction of deep coal-seam gas pressure and its application in coal mines. *Safety Science*.

[29] Sun ZX, Zhang W, Hu BQ, Li WJ, Pan TY (2005). The relationship between the geothermal field characteristics and the distribution of the CBM in the Quishui basin. *Chinese Science Bulletin*.

[30] Biot MA (1941). General theory of three-dimensional consolidation. *Journal of Applied Physics*.

[31] Detournay E, Cheng AHD, Fairhurst C (1993). Fundamentals of poroelasticity. *Comprehensive Rock Engineering: Principles, Practice and Projects, Vol. II, Analysis and Design Method*.

[32] Salmachi A, Haghighi M (2012). Feasibility study of thermally enhanced gas recovery of coal seam gas reservoirs using geothermal resources. *Energy and Fuels*.

[33] Do DD (1998). *Adsorption Analysis: Equilibria and Kinetics*.

[34] Harpalani S, Mitra A (2010). Impact of CO_2_ injection on flow behavior of coalbed methane reservoirs. *Transport in Porous Media*.

[35] Liu S, Harpalani S (2013). A new theoretical approach to model sorption induced coal shrinkage or swelling. *AAPG Bulletin*.

[36] Cui X, Bustin RM, Chikatamarla L (2007). Adsorption-induced coal swelling and stress: implications for methane production and acid gas sequestration into coal seams. *Journal of Geophysical Research B*.

[37] Karacan CÖ (2007). Swelling-induced volumetric strains internal to a stressed coal associated with CO_2_ sorption. *International Journal of Coal Geology*.

[38] Dawson GKW, Golding SD, Esterle JS, Massarotto P (2012). Occurrence of minerals within fractures and matrix of selected Bowen and Ruhr Basin coals. *International Journal of Coal Geology*.

[39] Chen Z, Pan Z, Liu J, Connell LD, Elsworth D (2011). Effect of the effective stress coefficient and sorption-induced strain on the evolution of coal permeability: experimental observations. *International Journal of Greenhouse Gas Control*.

[40] Pini R, Ottiger S, Burlini L, Storti G, Mazzotti M (2009). Role of adsorption and swelling on the dynamics of gas injection in coal. *Journal of Geophysical Research B*.

[41] McKee CR, Bumb AC, Koenig RA (1988). Stress-dependent permeability and porosity of coal and other geologic formations. *SPE Formation Evaluation*.

[42] Song Y, Zhang XM, Liu SB (2012). *The Basic Theory of Coalbed Methane Geology and Exploration in China*.

[43] Brown ET, Hoek E (1978). Trends in relationships between measured in-situ stresses and depth. *International Journal of Rock Mechanics and Mining Sciences and*.

[44] Tao S, Wang Y, Tang D (2012). Dynamic variation effects of coal permeability during the coalbed methane development process in the Qinshui Basin, China. *International Journal of Coal Geology*.

[45] Fu X, Qin Y, Jiang B, Wang W, Li G (2003). Physical and numerical simulations of permeability of coal reservoirs in central and southern part of the Qinshui Basin, Shanxi. *Scientia Geologica Sinica*.

[46] Yin G-Z, Jiang C-B, Xu J, Peng S-J, Li W-P (2011). Experimental study of thermo-fluid-solid coupling seepage of coal containing gas. *Journal of the China Coal Society*.

[47] Zhang SH, Tang SH, Wan Y, Li ZC, Zhang JP (2012). The Migration of CH_4_ and CO_2_ in jincheng anthracite coal. *Journal of China University of Mining and Technology*.

[48] Bangham DH, Franklin RE (1946). Thermal expansion of coals and carbonised coals. *Transactions of the Faraday Society*.

[49] Kelemen SR, Kwiatek LM (2009). Physical properties of selected block Argonne Premium bituminous coal related to CO_2_, CH_4_, and N_2_ adsorption. *International Journal of Coal Geology*.

